# Optimal scheduling strategy of electric vehicle based on improved NSGA-III algorithm

**DOI:** 10.1371/journal.pone.0298572

**Published:** 2024-05-17

**Authors:** Yun Wu, Du Yan, Jie-Ming Yang, An-Ping Wang, Dan Feng

**Affiliations:** 1 Department of Computer Science, Northeast Electric Power University, Chuanying District, Jilin, Jilin, China; 2 Scientific Research Industry Division, Northeast Electric Power University, Chuanying District, Jilin, Jilin, China; 3 Chaoyang Service Center of Ecology and Environment, Liaoning, China; SR University, INDIA

## Abstract

Aiming at the problem of load increase in distribution network and low satisfaction of vehicle owners caused by disorderly charging of electric vehicles, an optimal scheduling model of electric vehicles considering the comprehensive satisfaction of vehicle owners is proposed. In this model, the dynamic electricity price and charging and discharging state of electric vehicles are taken as decision variables, and the income of electric vehicle charging stations, the comprehensive satisfaction of vehicle owners considering economic benefits and the load fluctuation of electric vehicles are taken as optimization objectives. The improved NSGA-III algorithm (DJM-NSGA-III) based on dynamic opposition-based learning strategy, Jaya algorithm and Manhattan distance is used to solve the problems of low initial population quality, easy to fall into local optimal solution and ignoring potential optimal solution when NSGA-III algorithm is used to solve the multi-objective and high-dimensional scheduling model. The experimental results show that the proposed method can improve the owner’s satisfaction while improving the income of the charging station, effectively alleviate the conflict of interest between the two, and maintain the safe and stable operation of the distribution network.

## 1 Introduction

As a representative of renewable energy vehicles, electric vehicles are one of the important ways to achieve ’carbon peak and carbon neutrality’. In the future, large-scale access to the network will become an inevitable trend, but the problems such as severe load fluctuations and imbalance between supply and demand caused by them have brought great challenges to the planning and operation of power systems [1, 2]. At the same time, with the introduction of Vehicle-to-Grid (V2G) technology, EV can not only be used as a charging load, but also can be used as an energy storage power supply to realize the two-way flow of energy and reduce the power supply pressure of the power grid [3, 4]. Therefore, how to reasonably and effectively schedule the charging and discharging of electric vehicles, taking into account the needs of charging station operators and vehicle owners, and maintaining the safe and stable operation of the distribution network is an urgent problem to be solved.

In the scheduling of electric vehicles, the satisfaction of the owner will directly affect the enthusiasm of the owner to participate in the charging and discharging scheduling, and then affect the operation and development of the charging station [5, 6]. The owners respond to the charging station scheduling to reduce their own costs, but the benefits of the charging station will decrease while improving their own interests. Therefore, how to optimize the scheduling under the premise of ensuring the satisfaction of car owners and design a feasible scheduling scheme is an important problem to be solved in the electric vehicle scheduling market [7]. Huang Guihong et al. [8] constructed a double-layer charging and discharging model of electric vehicles considering wind power and owner satisfaction; While optimizing the scheduling, they reasonably took into account the owner satisfaction, but only considered the overall owner satisfaction and did not consider the single electric vehicle owner. Dong Yan et al. [9] adopted a two-tier scheduling strategy for electric vehicles, in the second stage of the lower-level model, the maximum satisfaction of the owner is the objective function, but the owner’s interests are ignored.

The rate and period of the traditional time-of-use electricity price are not dynamically adjusted with the actual system after the electric vehicle is connected to the network, and the dynamic characteristics of the EV are not considered. So in recent years, the electricity price mechanism has received extensive attention from researchers as an indirect method to control electric vehicles participating in charge and discharge scheduling [10]. Chen Lupeng et al. [11] proposed a real-time scheduling model for large-scale electric vehicles considering the interests of multiple electric vehicle aggregators (EVA); By considering the load rate of transformers in each period of time, the dynamic electricity price is formulated. However, in the process of electric vehicle discharge, there is no corresponding strategy and measures to respond to the fluctuation of electricity prices in different time periods. Cui Jindong et al. [12] analyzed the charging and discharging pricing model of electric vehicles from multiple perspectives, and proposed win-win for multiple parties pricing strategy to guide the orderly charging and discharging of electric vehicles; However, they ignored the willingness of the owner side to participate in the Vehicle-to-Grid (V2G) scheduling. Zhang Xizhu et al. [13] proposed a hierarchical scheduling strategy for electric vehicles under the dynamic time-of-use electricity price mechanism; The dynamic time-of-use electricity price is formulated by the real-time updated load forecasting curve of the distribution system, but only the economic benefits of a single participant are considered.

Based on the above analysis, In order to better balance the conflict of interest between the electric vehicle charging station and the owner and slow down the load fluctuation, this paper proposes an electric vehicle scheduling model considering the comprehensive satisfaction of the owner. Aiming at the problems that the initial population of the NSGA-III algorithm cannot adapt to the dynamic characteristics of electric vehicles entering the network, is prone to getting stuck in local optimal solutions, and has poor population diversity in the process when solving the multi-objective model, an improved NSGA-III algorithm (DJM-NSGA-III) is proposed.

## 2 Analysis of charging and discharging capacity of electric vehicle

The remaining available power of the electric vehicle battery is a key factor affecting whether the electric vehicle can participate in the charging and discharging scheduling. If the remaining available power of the battery is greater than the threshold and ensures that the scheduling meets the travel requirements of the owner when the owner is expected to complete the charging time, the electric vehicle can participate in the charging and discharging scheduling. Therefore, before the charging and discharging scheduling of electric vehicles, it is necessary to fully study the state of charge of electric vehicle batteries to ensure the safety and feasibility of charging and discharging operations.

The service life of the lithium battery is related to its discharge depth, which refers to the percentage of the amount of electricity used by the lithium battery during discharge to its rated capacity. When the discharge depth of lithium battery exceeds a certain extent, it will lead to the aggravation of lithium battery loss, thus shortening its service life. Studies have shown that when the discharge depth is 80%, the service life is about 3000 times [14]. Therefore, this paper sets 0.2 as the threshold. When the state of charge of the battery when the electric vehicle arrives at the station is greater than 0.2, it participates in the discharge scheduling. It preferentially discharges until the state of charge is equal to 0.2 and stops discharging, and then participates in the charging scheduling. When the state of charge of the battery when the electric vehicle arrives at the station is less than 0.2, it is preferred to participate in the charging scheduling.

The initial state of charge of the electric vehicle is shown in Formula (1) [15]:

$$SOC=SOC_{e}-\frac{TP\partial }{Q}$$ (1)

In the formula: *SOC* represents the initial state of charge when the electric vehicle reaches the charging station; *SOC*_*e*_ represents the owner’s expected state of charge; *Q* represents the rated capacity of the battery; *T* represents the charging time; *P* represents the charging power of electric vehicles; ∂ represents the charging efficiency.

## 3 Electric vehicle scheduling model considering the comprehensive satisfaction of vehicle owners

In order to guide the owner to actively participate in the scheduling, alleviate the conflict of interest between the charging station and the owner, and make the charging station achieve a balance between the operating income and the system health, this paper comprehensively considers the factors such as the economy of the charging station, the interest demand of the owner and the load fluctuation of the electric vehicle, and takes the dynamic electricity price and the charging and discharging state of the electric vehicle as the decision variables. The charging and discharging scheduling model of the electric vehicle is established with the goal of maximizing the comprehensive satisfaction of the owner ’s economic interests, maximizing the income of the charging station and minimizing the peak-valley difference rate of the electric vehicle load.

### 3.1 Charging station revenue model

The electric vehicle charging station is modeled with the goal of maximizing revenue, and its mathematical expression is shown in Formula (2) [16]:

maxF1=∑t=1T∑n=1N(C(t)Pn+C(t)Pn') (2)

In the formula: *F*_1_ represents the income of the charging station; *T* represents the number of time periods, and the day is divided into 24 time periods at an interval of 1h; *t* represents the *t* period; *N* represents the number of electric vehicles; *C*_(*t*)_ represents the dynamic electricity price of the *t* period; *P*_*n*_ represents the charging power of the *n*th electric vehicle; Pn' represents the discharge power of the *n*th electric vehicle, which is negative.

### 3.2 Owner comprehensive satisfaction model

Owner satisfaction will directly affect the enthusiasm of owners to participate in the charging and discharging scheduling of electric vehicle charging stations, thus affecting the operation and development of charging stations. In order to balance the contradiction between the operating income of charging stations and owner satisfaction, this paper comprehensively considers the needs of owners own interests, proposes owner economic satisfaction, and combines owner travel demand satisfaction and electric vehicle battery loss satisfaction to build a owner comprehensive satisfaction model.

The charging and discharging cost of electric vehicles is a key factor affecting the owner economic satisfaction. Therefore, the owner economic satisfaction is proposed as shown in Formula (3):

αn1=min{1−∑t=1T[(C(t)Pn−C(t)Pn')−(CminPn−CmaxPn')]∑t=1T[(CmaxPn−CminPn')−(CminPn−CmaxPn')],1} (3)

In the formula: *α*_*n*1_ represents the economic satisfaction of the *n*th electric vehicle owner; *C*_max_ represents the upper limit of dynamic electricity price; *C*_min_ represents the lower limit of dynamic electricity price; *C*_(*t*)_ represents the dynamic electricity price of *t* period. Electric vehicles are arranged to charge during the valley period of electricity price as much as possible, and discharge during the peak period of electricity price. The less the owner spends, the higher the economic satisfaction is. When the electric vehicle is discharged during the maximum price period and charged during the minimum price period, the owner economic satisfaction reaches the maximum value of 1.

The owner’s charging demand satisfaction is directly related to the state of charge of the electric vehicle, as shown in Formula (4) [17]:

$$\alpha_{n2}=\min\{S_{f,n,e}/S_{f,n,\exp},\;1\}$$ (4)

In the formula: *α*_*n*2_ represents the owner’s charging demand satisfaction; *S*_*f*,*n*,*e*_ represents the state of charge of the electric vehicle when the owner reaches the charging station; *S*_*f*,*n*,exp_ represents the state of charge when the owner expects to leave. When the owner leaves the charging station, the closer the state of charge of the electric vehicle is to the expected state of charge, the more it can meet the owner’s travel needs, the higher the satisfaction, and the ideal state of charge is 1.

In the process of charging and discharging scheduling, the electric vehicle battery charging and discharging conversion will be lost, and its satisfaction is shown in Formula (5):

$$\alpha_{n3}=\min\{1/(1+\eta_{n,ch\arg e}),\;1\}$$ (5)

In the formula: *α*_*n*3_ represents the battery loss satisfaction; *η*_*n*,*ch*arg*e*_ represents the number of charge and discharge conversions of the *n*th electric vehicle during the scheduling process. In the process of electric vehicle scheduling, the less the number of battery charge and discharge conversions, the higher the satisfaction, and the maximum can be taken to 1.

Combining the owner economic satisfaction *α*_*n*1_, the owner charging demand satisfaction *α*_*n*2_ and the electric vehicle battery loss satisfaction *α*_*n*3_, the model is established with the goal of maximizing the owner comprehensive satisfaction, as shown in Formula (6):

$$\max F_{2}=\frac{1}{N}\sum_{n=1}^{N}\alpha_{n1}\alpha_{n2}\alpha_{n3}$$ (6)

In the formula: *F*_2_ represents the owner’s comprehensive satisfaction and its value range is [0,1]. The closer it is to 1, the greater the satisfaction is, the higher the willingness to participate in the scheduling is.

### 3.3 Electric vehicle load fluctuation model

In the case of disorderly charging of electric vehicles, a new load peak will be formed, which will affect the stable operation of the distribution network. Therefore, the charging and discharging scheduling of the charging station hopes to use the energy storage characteristics of the electric vehicle to feed back the electric energy to the grid through the battery [18, 19]. Therefore, in order to make the power system better respond to load fluctuations, reduce the impact of electric vehicle access on the distribution network, and improve the reliability and stability of the power grid, a model is established with the goal of minimizing the peak-to-valley difference of electric vehicle charging and discharging load, as shown in Formula (7):

$$\min F_{3}=\frac{P_{\max}-P_{\min}}{P_{\max}}$$ (7)

In the formula: *F*_3_ represents the peak-valley difference rate of electric vehicle load; *P*_max_ represents the maximum charge and discharge power of electric vehicle; *P*_min_ represents the minimum charge and discharge power of the electric vehicle.

Therefore, the optimal scheduling model of electric vehicles is shown in Formula (8):

$$\max F={\left\{\max F\right\}}_{1},\max F_{2},-\min F_{3}\}$$ (8)

### 3.4 Constraint condition

#### (1) Battery state of charge constraint

In order to prevent the excessive discharge of electric vehicles from causing loss to the battery, the state of charge of the electric vehicle battery is constrained to ensure the rights and interests of owners, as shown in Formula (9) [20]:

$$SOC_{\min,t}^{n}\leq SOC_{t}^{n}\leq SOC_{\max,t}^{n}$$ (9)

In the formula: $SOC_{t}^{n}$ represents the state of charge of the *n*th electric vehicle in the *t* period; $SOC_{\max,t}^{n}$ represents the maximum value of the state of charge of the *n*th electric vehicle in the *t* period; $SOC_{\min,t}^{n}$ represents the minimum state of charge of the *n*th electric vehicle in the *t* period.

#### (2) Electric vehicle charging and discharging state constraints

The electric vehicle can only be in one of the three states of charging, discharging and neither charging nor discharging at each time period. Therefore, the charging and discharging state of the electric vehicle is constrained, as shown in Formula (10):

$$P_{n}\times P_{n}^{'}=0$$ (10)

In the formula: *P*_*n*_ represents the charging power of the *n* th electric vehicle; *P_n_*' represents the discharge power of the *n* th electric vehicle, which is negative.1

#### (3) Electric vehicle charging and discharging scheduling time constraints

The charging behavior of electric vehicles is random, so the schedulable time period should be from the time when the electric vehicle arrives at the charging station to the time when it leaves the charging station, as shown in formula (11):

$$t_{s}<t'<t_{e}$$ (11)

In the formula: *t*' is the schedulable time of electric vehicles; *t*_*s*_ and *t*_*e*_ are the time when the electric vehicle arrives at the charging station and leaves the charging station, respectively.

## 4 Using common multi-objective optimization algorithms for solving

**Fig 1:**
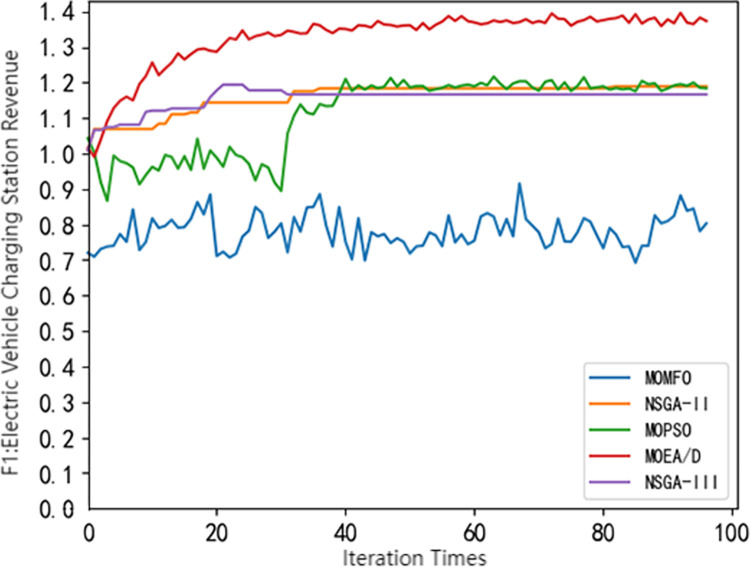
Pareto frontier comparison of different algorithms(*F*_1_).

**Fig 2:**
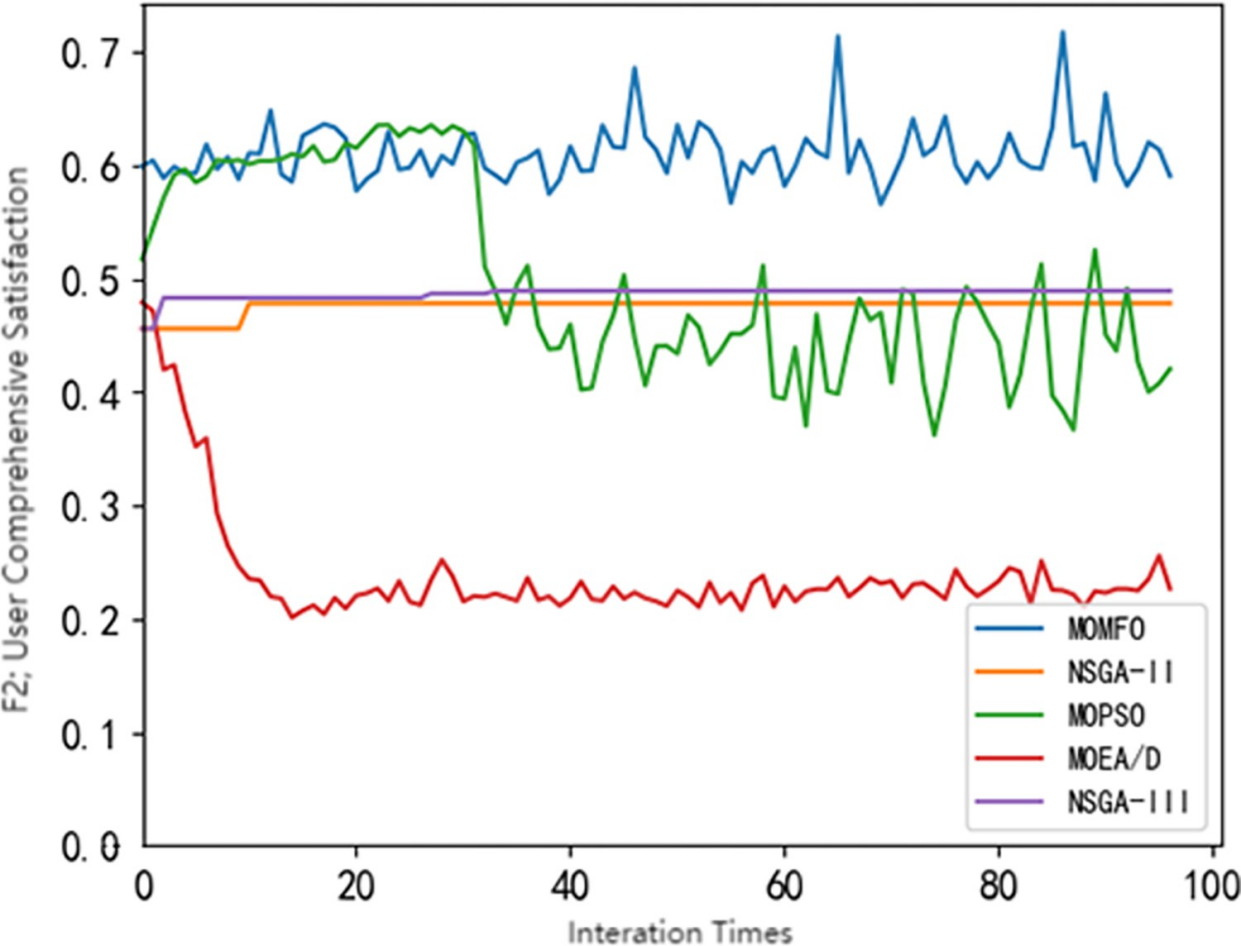
Pareto frontier comparison of different algorithms(*F*_2_).

**Fig 3:**
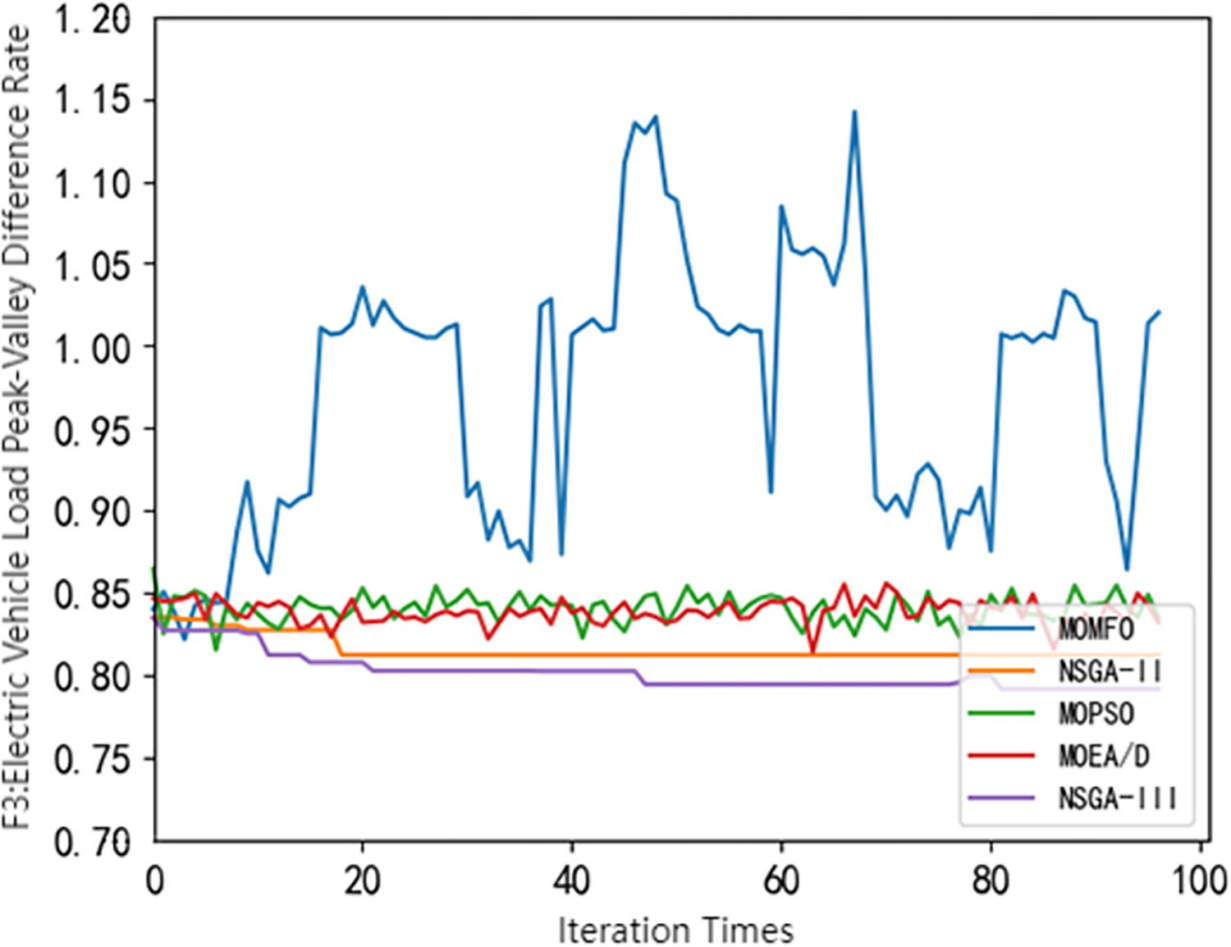
Pareto frontier comparison of different algorithms(*F*_3_).

**Fig 4:**
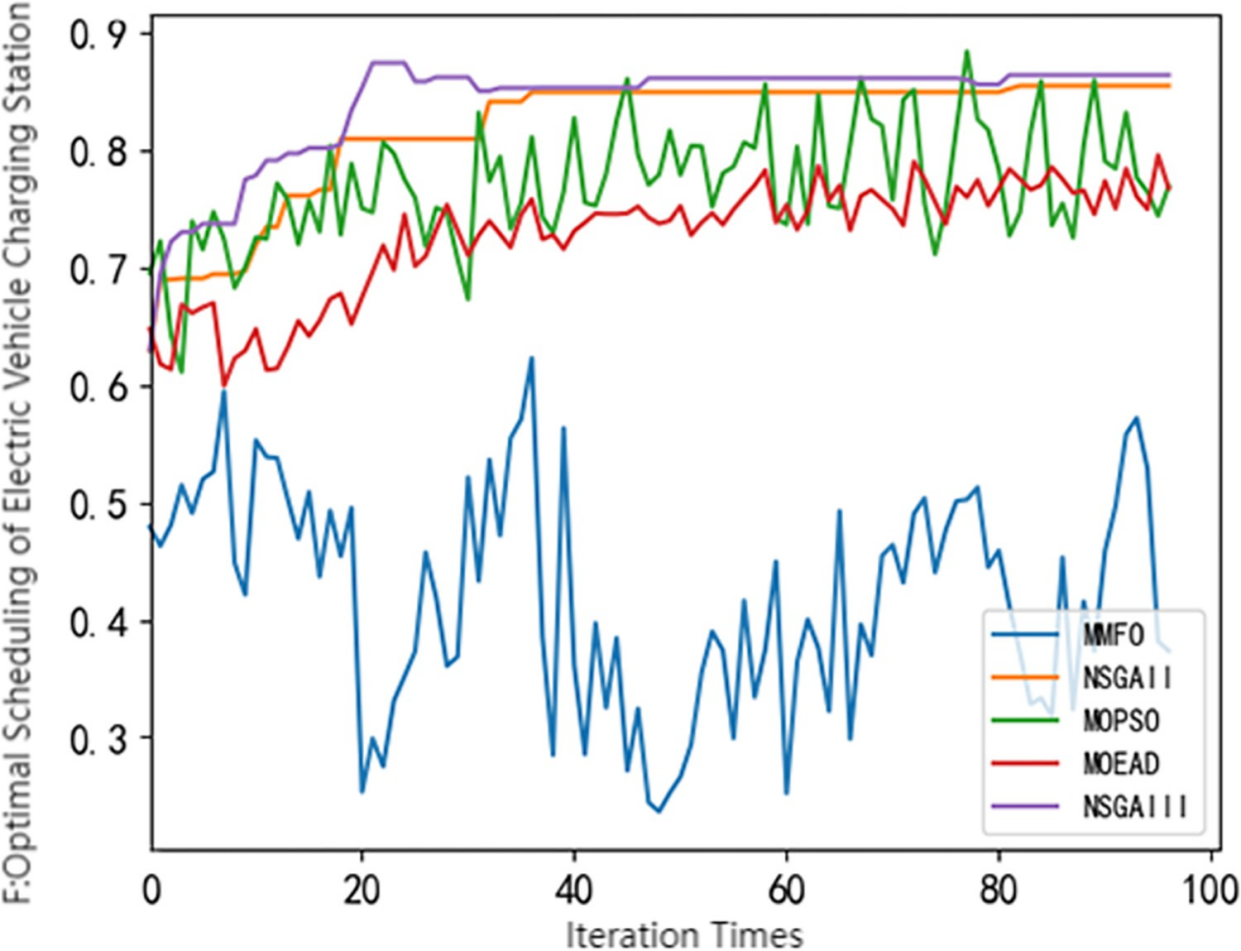
Pareto frontier comparison of different algorithms(*F*).

The optimization scheduling of electric vehicles belongs to the classic multi-objective optimization problem, and the scheduling strategy needs to measure the satisfaction of vehicle owners, the revenue of electric vehicle charging stations, and the fluctuation of electric vehicle loads from multiple aspects. This article adopts five representative optimization algorithms in the field of multi-objective optimization, namely MOEA/D, NSGA-II, NSGA-III, MOMFO, and MOPSO algorithms. These algorithms are used to optimize and solve the electric vehicle scheduling model in this article, The results are shown in Figs 1–4:

It can be seen from Figs 1–4 that different swarm intelligence algorithms focus on different objective functions. The MOEA/D algorithm shows good optimization characteristics in maximizing the revenue of electric vehicle charging stations. The MOMFO algorithm focuses more on the goal of maximizing the comprehensive satisfaction of car owners; the optimization ability of the MOPSO algorithm on the three objective functions is at a medium level; the optimization ability of NSGA-II algorithm and NSGA-III algorithm on the target of electric vehicle charging station side and vehicle owner side is above the average level, and more emphasis is placed on the optimization target of electric vehicle load. Among them, NSGA-III algorithm has better optimization ability in minimizing the peak-valley difference rate of electric vehicle load than NSGA-II algorithm. It can be seen from Fig 4 that the NSGA-III algorithm shows excellent performance in optimization, which is not only superior to other algorithms, but also has excellent stability. Based on the above results, it shows the significant potential and superiority of the NSGA-III algorithm in solving the problem of electric vehicle charging station scheduling strategy, but there are also some defects. Therefore, this paper uses the improved NSGA-III algorithm to solve the model.

## 5 DJM-NSGA-III algorithm

**Fig 5:**
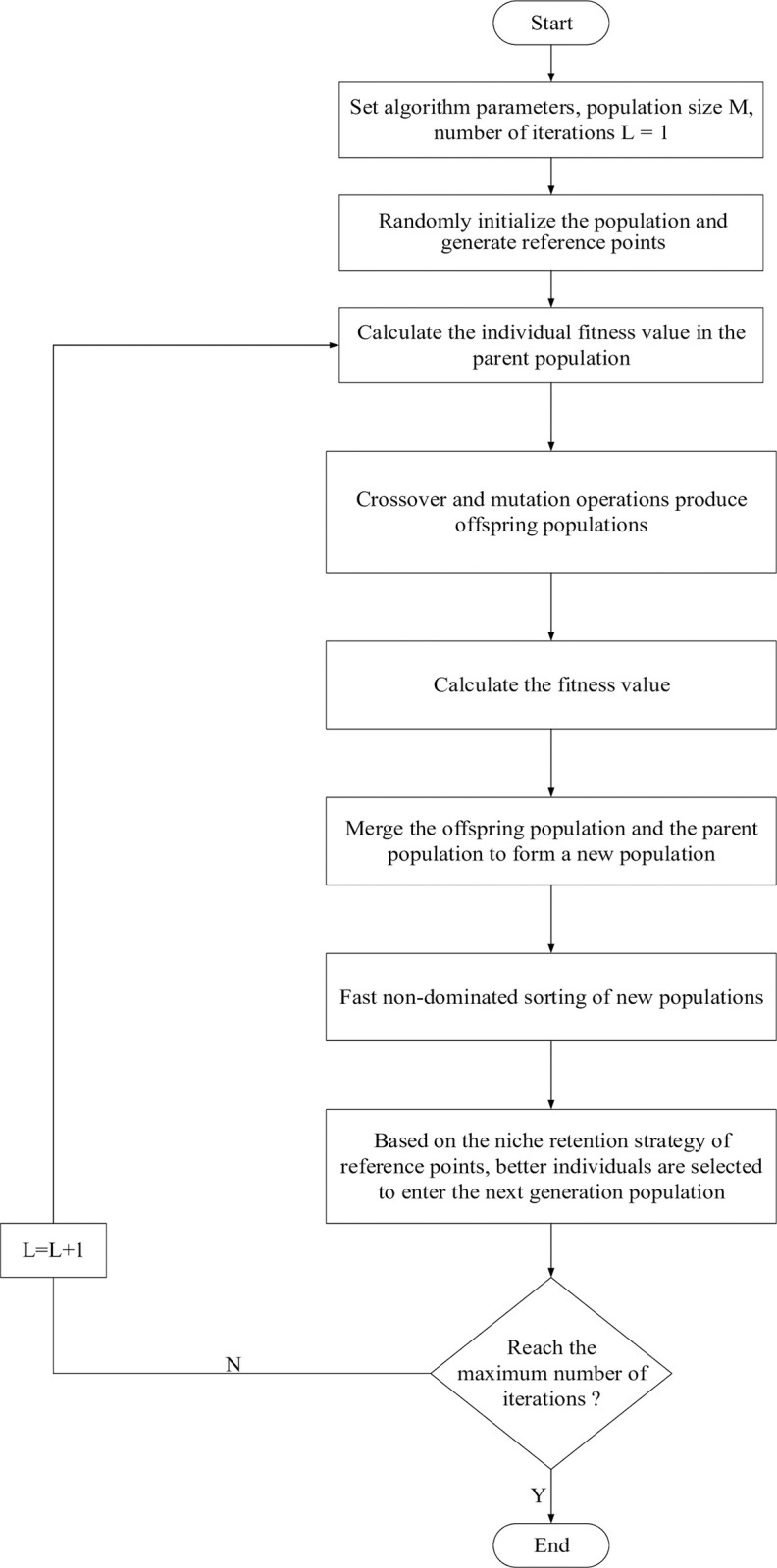
Flow chart of NSGA-III algorithm.

Deb proposed the NSGA-III algorithm in 2012, which is an extension of the NSGA-II algorithm. The non-dominated sorting technique is used to classify the scheduling strategies into different frontiers according to their dominant relationships. The crowding degree is used to maintain the diversity in each frontier, and the reference point selection method is introduced to make the population have a good distribution. The basic flow chart is shown in Fig 5.

When the traditional NSGA-III algorithm is used to solve the multi-variable, multi-objective and high-dimensional optimization model, the initial population cannot adapt to the dynamic characteristics of electric vehicles entering the network, it is easy to fall into the local optimal solution, and the population diversity in the process is poor. Therefore, the DJM-NSGA-III algorithm is proposed based on dynamic opposition-based learning strategy, improved Jaya algorithm and Manhattan distance.

### 5.1 Using dynamic opposition-based learning strategy initialization

In order to ensure the dispersion of the population in the search space, the original NSGA-III algorithm uses a random generation strategy for initialization. Although it helps to increase the exploration range of the solution space, it cannot guarantee the quality of the initial population and is easy to lose diversity. The individuals in the initial population may be concentrated in certain areas, resulting in poor distribution of the Pareto front. The algorithm converges locally in the solution space and misses the potential global optimal solution. Therefore, this paper proposes to apply the dynamic opposition-based learning strategy to the initialization of the NSGA-III algorithm. By distributing individuals in the asymmetric dynamic search space, the quality of the initial solution is improved and the probability of finding the global optimal solution is improved [21]. The initialization is shown in formula (12):

$$X_{DOBL}=X_{init}+r_{1}\times (r_{2}\times (LB+UB-X_{init})-X_{init})$$ (12)

In the formula: *X*_*DOBL*_ represents the reverse initial population; *X*_*init*_ represents the original initialization population; *r*_1_ and *r*_2_ are random numbers of 0~1; *LB* and *UB* are the upper and lower boundaries of the population. The algorithm combines *X*_*DOBL*_ and *X*_*init*_ into a new population, calculates the fitness value of the new population, and uses the greedy strategy to fully compete within the population, and selects the best individual as the initial population.

### 5.2 Mutation operation

The mutation operation of the traditional NSGA-III algorithm adopts single point mutation. With the iteration of the algorithm, the population is easy to evolve in the wrong direction, thus falling into the local optimum. In the search of electric vehicle charging and discharging strategy, considering that a large number of excellent populations will be generated in the later stage of the search, it is necessary to pay attention to local search and optimize the population quality. Therefore, in order to solve the problem of uneven global search ability and local search ability of the algorithm, the Jaya optimization algorithm is used in the mutation operation [22]. Based on the original Jaya optimization algorithm, the difference between the individual and the current population average is proposed to help maintain the diversity of the population. Based on the principle of continuous improvement, the individual is constantly moving closer to the excellent individual and away from the poor individual. The search focuses on the global search in the early stage. With the increase of the number of iterations, the later search performs local search in the excellent individual to improve the search accuracy and accelerate convergence. The mutation operation based on the improved Jaya optimization algorithm proposed in this paper is shown in Formula (13):

$$Y{'}_{k,m,l}=Y_{k,m,l}+r_{best,k,m,l}\times (Y_{best,k,m,l}-|Y_{k,m,l}|)-r_{worst,k,m,l}(Y_{worst,k,m,l}-|Y_{k,m,l}|)+r_{avg,k,m,l}(|Y_{k,m,l}|-Y_{avg,k,m,l})$$ (13)

In the formula: *k* represents the *k*th individual in the population; *m* represents the *m*th dimension variable of the individual; *l* represents the current number of iterations; *r*_*best*,*k*,*m*,*l*_, *r*_*worst*,*k*,*m*,*l*_ and *r*_*avg*,*k*,*m*,*l*_ are random parameters between [0,1]; *Y*_*k*,*m*,*l*_ and *Y*'_*k*,*m*,*l*_ represent the values of the *k*th individual in the *l*th generation of the first generation before the *m*-dimensional variation and after the Jaya variation; *Y*_*best*,*k*,*m*,*l*_ and *Y*_*worst*,*k*,*m*,*l*_ represent the historical optimal subpopulation and the worst subpopulation at the *l* th iteration, respectively; *Y*_*avg*,*k*,*m*,*l*_ represents the mean value of the subpopulation at the *l*th iteration.

### 5.3 Improving elite selection strategies

The NSGA-III algorithm uses a fast non-dominated sorting and reference point selection strategy to screen the recombinant population. The elite retention strategy will select the non-dominated optimal solution generated by each generation into the next generation population, so that all individuals will quickly move closer to the Pareto frontier region. However, this will lead to a high degree of similarity in the individual quality of the next generation, reduce the diversity in the population process, ignore the effective information carried by the infeasible solution, and overemphasize the feasibility. Therefore, in order to fully search for potential optimal solutions and improve the diversity of solutions, the elite selection strategy is improved. The specific steps are as follows:

1. According to the rapid non-dominated sorting, all individuals in the new population *R*_*t*_ formed by merging the parent and offspring populations are divided into several non-dominated layers such as grade *F*_1_,*F*_2_,*F*_3_,⋯,*F*_*n*_;
2. Starting from the *F*_1_ layer, it is selected to be retained in the population *S*_*t*_ in turn. When the individual size in *S*_*t*_ is greater than or equal to *M*, it is stopped, and the current non-dominated layer is *F*_*g*_. If the population size in *S*_*t*_ is equal to *M* at this time, the iteration ends, and *S*_*t*_ is used as the parent population *P*_*t*+1_ of the next iteration. If the population size in *S*_*t*_ is larger than *M*: ①When *g*>1, the individuals of the non-dominated layer before *F*_*g*_ are retained in the parent population *P*_*t*+1_ of the next iteration, and the individuals of the non-dominated layer before *F*_*g*_ are removed from *R*_*t*_ to form population *S*'_*t*_, and the Manhattan distance between all individuals in population *S*'_*t*_ is calculated. For any individual *k*, find out the other individuals closest to *k*, and calculate the sum of the Manhattan distance with *k* individuals, denoted by *SUM*. According to the value of individual *SUM*, select individuals from large to small and put them into *P*_*t*+1_ until the population reaches *M*. ②When *g* = 1, through the niche retention strategy of the reference point, several better individuals in *F*_1_ are selected to be retained in *P*_*t*+1_ until the population reaches *M*.

**Fig 6:**
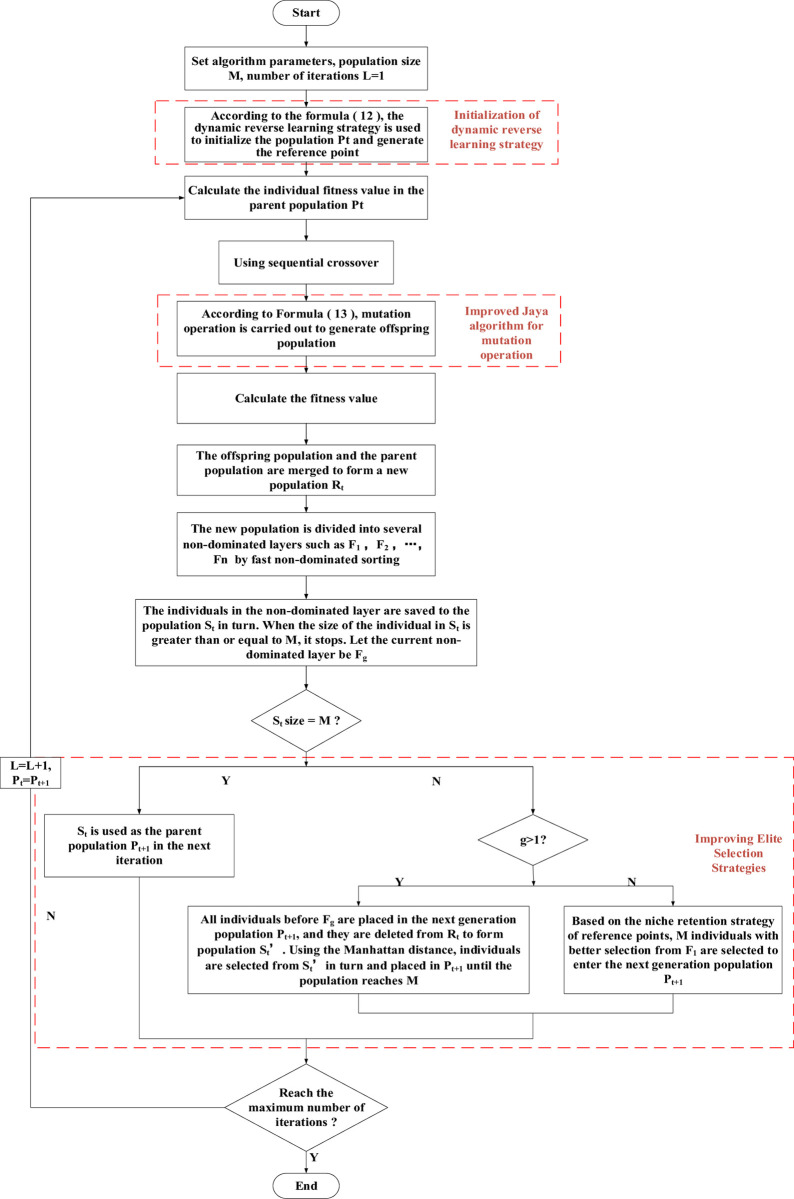
Flow chart of DJM-NSGA-III algorithm.

The DJM-NSGA-Ill algorithm, flowchart is shown in Fig 6:

## 6 Example analysis

### 6.1 Simulation parameters

This paper selects the data collected by California charging stations. These data cover two main categories of charging stations, namely park charging stations and public charging stations in surrounding residential areas. In these data, park charging stations occupy the main part. The probability density function is obtained by fitting the charging and discharging behavior of the electric vehicle’s initial charging time, daily mileage and charging end time. The Monte Carlo method is used to randomly select the probability density function corresponding to the charging and discharging behavior of the vehicle’s owner. The 24 hours of a day is divided into 24 periods, and the disordered charging and discharging load of the electric vehicle is predicted.

The number of simulated electric vehicles is 1,000. According to the common electric vehicles in the market, the parameters are set as follows: battery capacity is 82 kW · h, conventional charging power is 7 kW, charging efficiency is 0.9, and power consumption per 100 km is 20.5 kW · h. The time-of-use price is as shown in Table 1 [23]. The charging station service fee is charged according to the time-of-use price of the power grid in a 5:3:1 manner.

**Table 1:** Time-of-use electricity price table for each period.

time interval	Power grid time-of-use electricity price / (yuan / (kw · h))	Charging station time-of-use electricity price / (yuan / (kw · h))
00:00–08:00	0.365	0.4
08:00–12:00	0.869	2.0
12:00–15:00	0.687	1.2
15:00–17:00	0.687	2.0
17:00–21:00	0.869	2.0
21:00–24:00	0.687	1.2

### 6.2 Algorithm performance

In order to verify the effectiveness of the DJM-NSGA-III algorithm, this paper selects the original NSGA-III algorithm, R-NSGA-III algorithm [24] and U-NSGA-III algorithm [25]as the comparison algorithm to optimize the electric vehicle optimization scheduling model.

**Fig 7:**
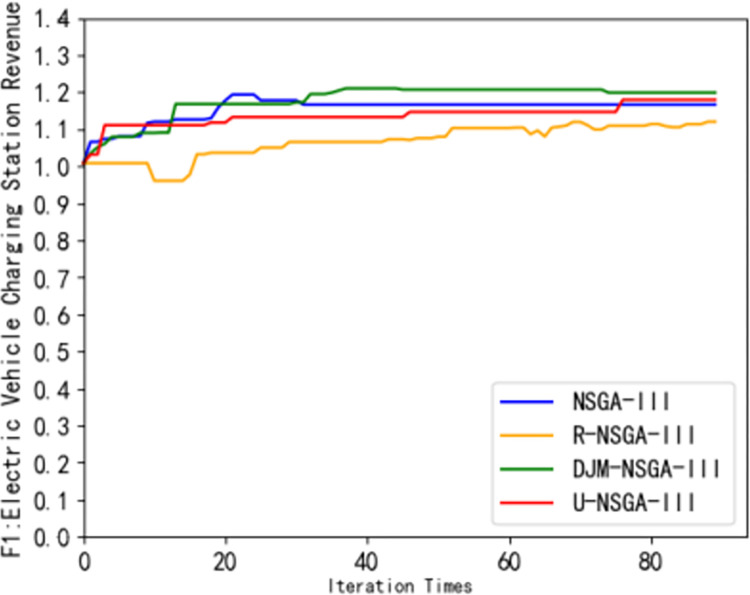
Comparison of Pareto frontiers of different algorithms (*F*_1_).

**Fig 8:**
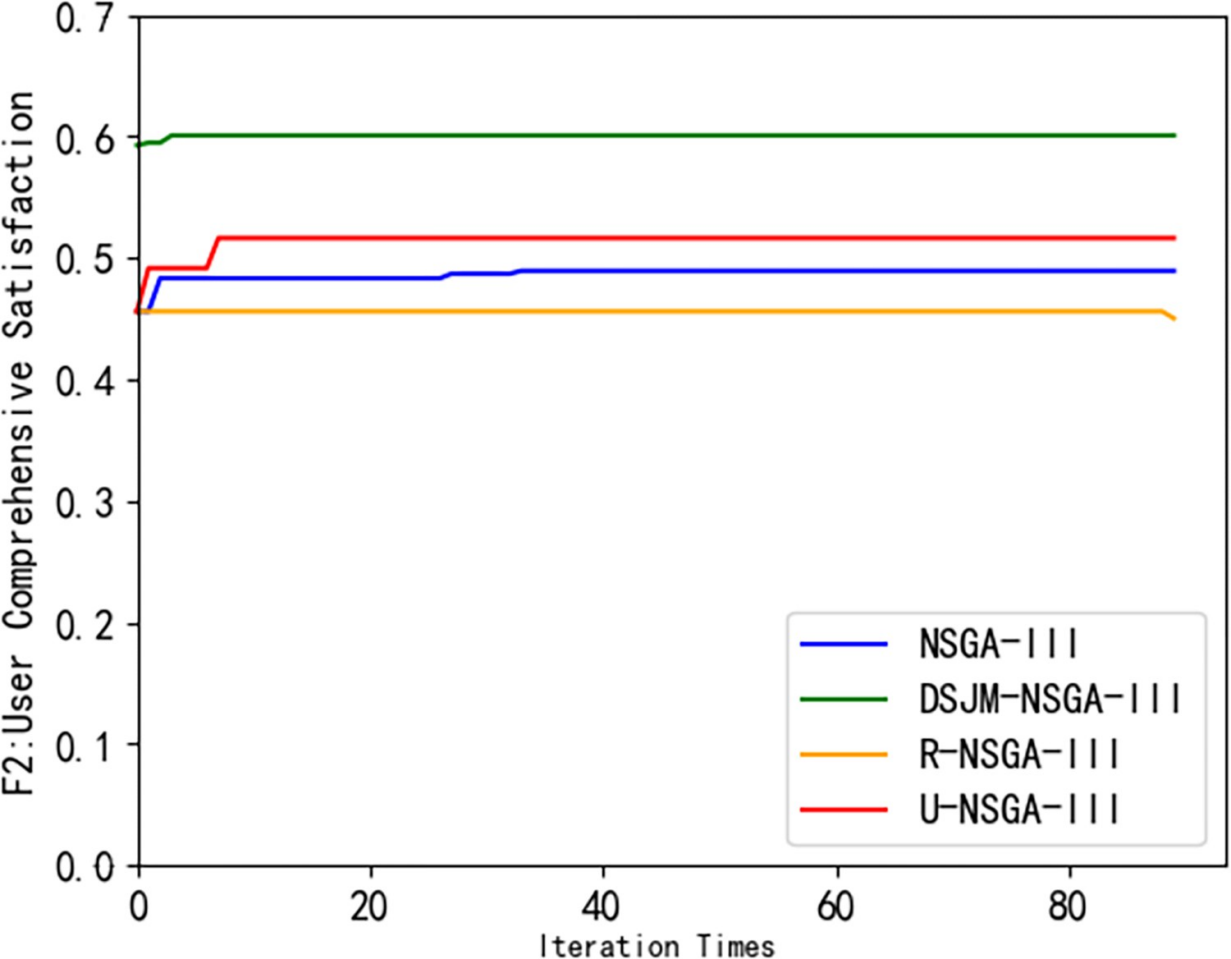
Comparison of Pareto frontiers of different algorithms (*F*_2_).

**Fig 9:**
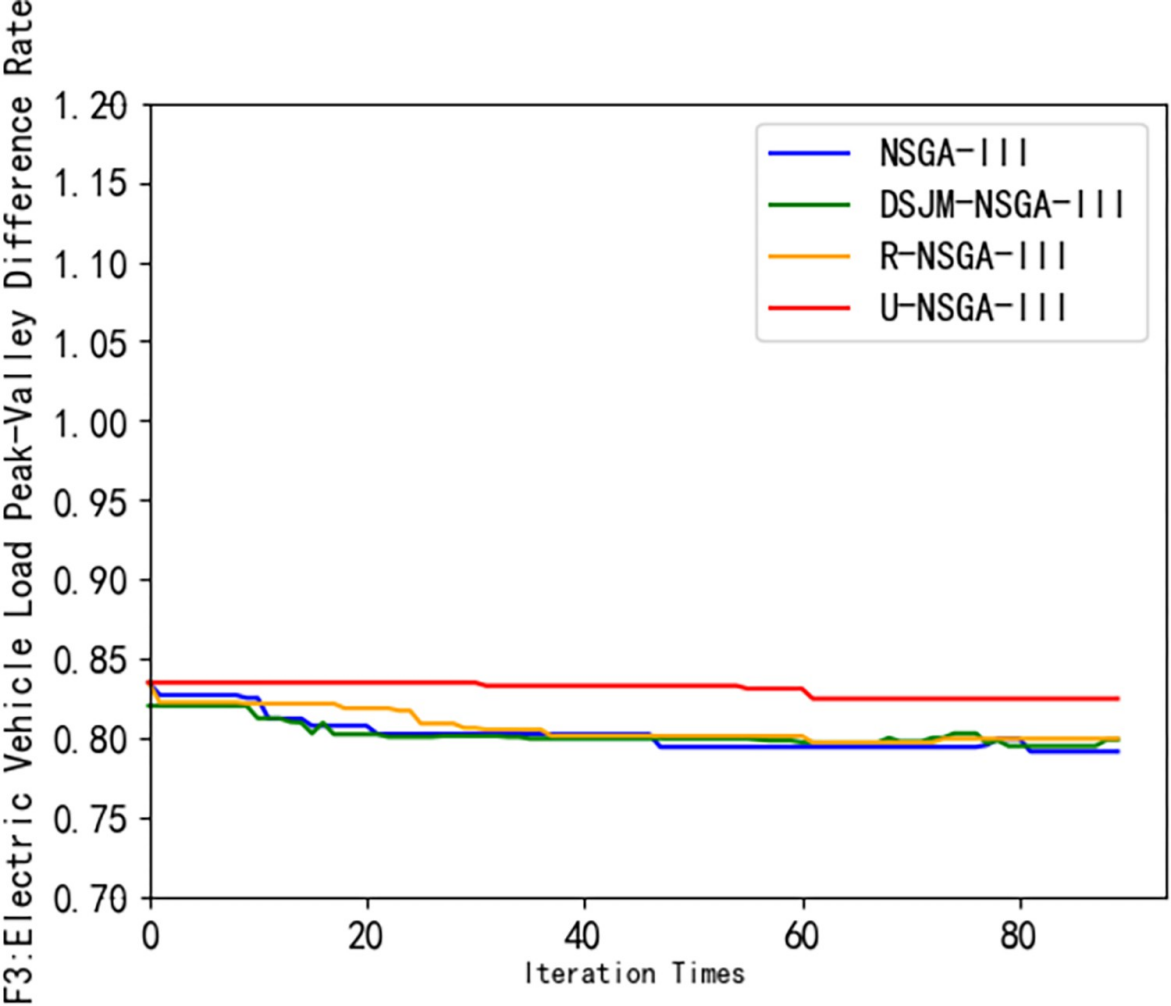
Comparison of Pareto frontiers of different algorithms (*F*_3_).

It can be seen from Fig 7 that the DJM-NSGA-III algorithm converges and finds the optimal solution at the average 39th iteration, and the revenue of the charging station is higher than that of other algorithms. It can be seen from Fig 8 that the owner’s satisfaction under the DJM-NSGA-III algorithm is higher than other algorithms. The curve is smoother and has always been at the top. The optimal solution is found at the average fourth iteration, indicating that the DJM-NSGA-III algorithm is more stable. It can effectively search for the global optimal solution and has a better Pareto solution. U-NSGA-III finds the average optimal solution at the average 9th iteration. Although it has jumped out of the local optimal solution, the effect is limited. NSGA-III finds the optimal solution at the average second iteration. Although the convergence speed is fast, it is easy to fall into the local optimal solution. The R-NSGA-III curve is almost horizontally at the bottom of the image. Although it has strong convergence ability, it has insufficient ability to jump out of local optimum. In Fig 9, the convergence speed of DJM-NSGA-III algorithm is faster than that of traditional NSGA-III. The load peak-valley difference rate of DJM-NSGA-III algorithm is slightly lower than that of other algorithms before about 35 iterations. After 35 iterations, the load peak-valley difference rate of NSGAIII, R-NSGA-III and DJM-NSGA-III is similar, which is lower than that of U-NSGA-III.

**Fig 10:**
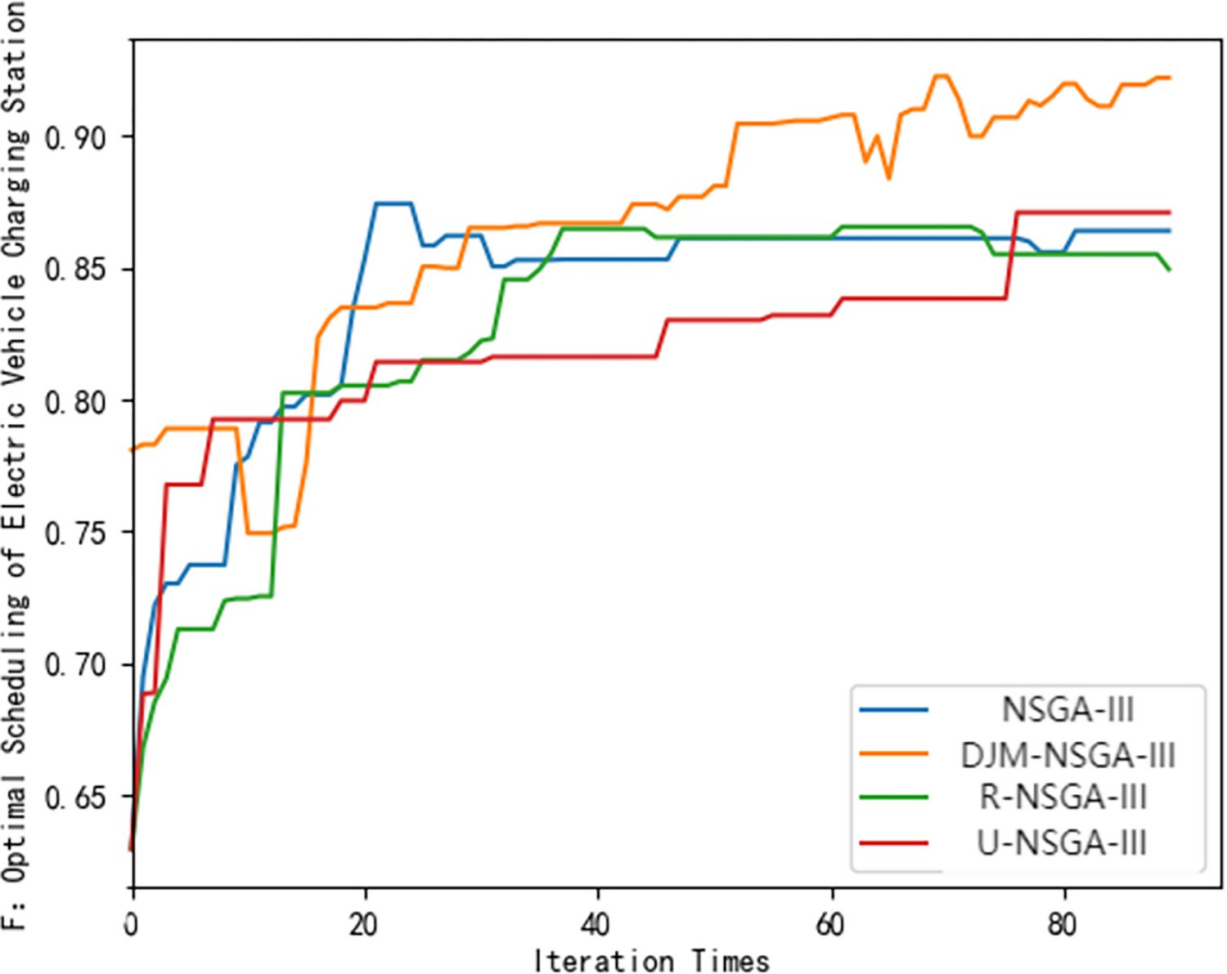
Comparison of Pareto frontiers of different algorithms (*F*).

Fig 10 shows the Pareto frontier generated by using different algorithms to solve the optimal scheduling model of electric vehicles. It can be seen that the dynamic opposition-based learning strategy adopted by DJM-NSGA-III algorithm makes the initial population quality better than other algorithms, and can maintain good population diversity in the subsequent optimization process. In addition, the Pareto solution obtained by the DJM-NSGA-III algorithm is also significantly better than other algorithms, so that it can jump out of the local optimal solution limit and fully search for the potential optimal solution in the later stage of iteration.

It can be seen from Table 2 that the number of convergence iterations required by the R-NSGA-III algorithm is at least 75 times, and the average generation value of DJM-NSGA-III is 0.87. Although the number of convergence iterations of the DJM-NSGA-III algorithm is slightly higher than that of the original algorithm, the average generation value is improved, which shows the superiority of the DJM-NSGA-III algorithm in solving the scheduling strategy problem of electric vehicle charging stations.

**Table 2:** Performance comparison of different algorithms.

Algorithm	Average convergence iteration times	Average generation value
DJM-NSGA-III	88	0.87
NSGA-III	80	0.81
U-NSGA-III	76	0.80
R-NSGA-III	75	0.81

### 6.3 Analysis of model results

**Fig 11:**
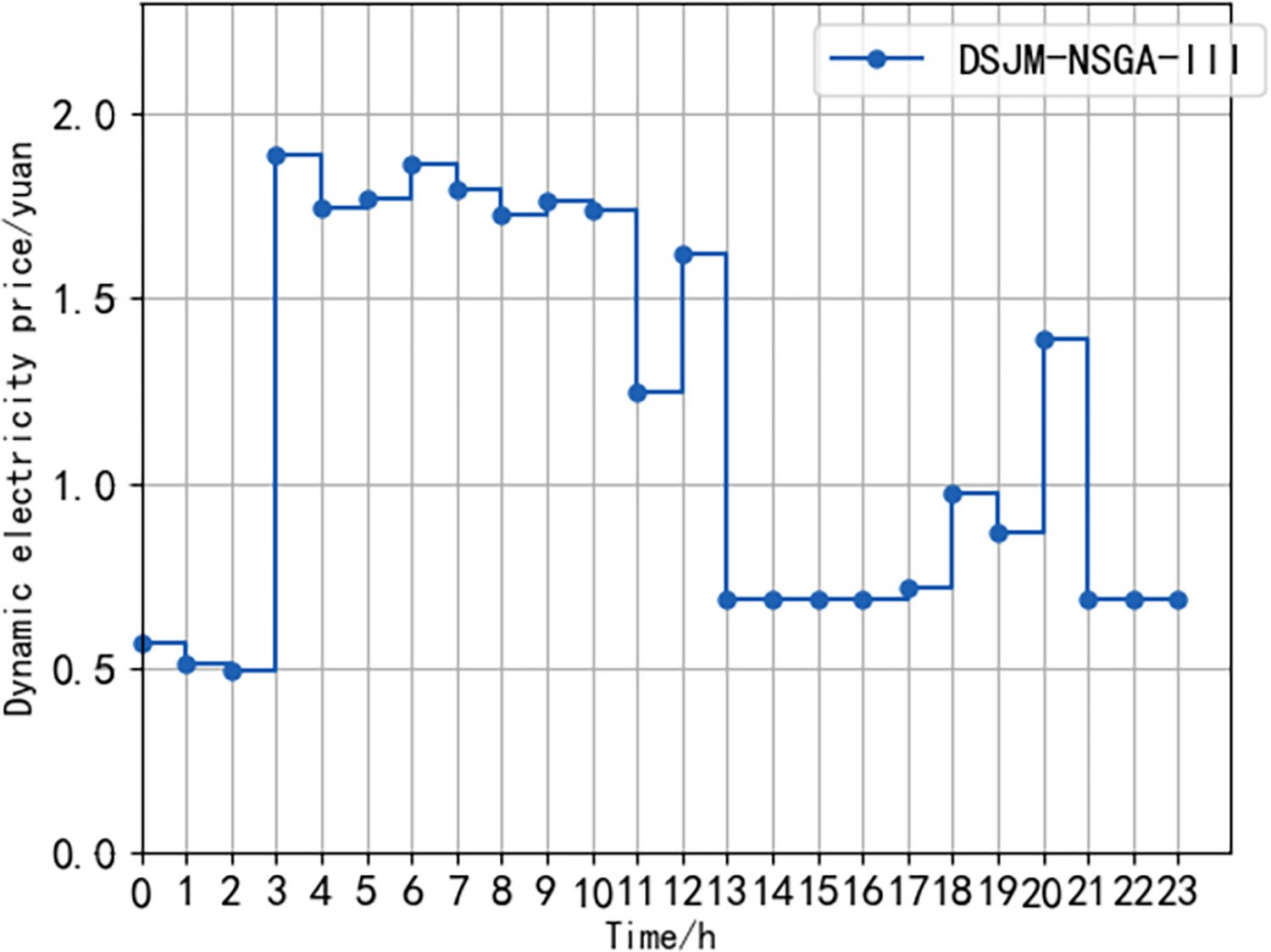
Charging station dynamic price diagram of each period.

Fig 11 is the dynamic electricity price of the charging station obtained by using the DJM-NSGA-III algorithm to optimize the scheduling of electric vehicles. The overall trend shows that 03:00–13:00 is the peak period of electricity price. This is because between 03:00 and 6:00, the park staff has not yet started to work. The main body of these periods is the surrounding residents’ electric vehicles. In order to improve their own interests, the charging station chooses to increase the dynamic electricity price. After 6:00, as the employees go to work, the number of electric vehicles increases rapidly, and the overall load also increases. Therefore, in order to alleviate the load pressure after work, a high dynamic electricity price is maintained between 6:00 and 13:00, which guides the electric vehicle to discharge at this time, avoids the sharp load fluctuation caused by concentration, feeds the distribution network and disperses the charging demand. Between 13:00 and 20:00, it is in the valley period of electricity price. This is because in order to meet the travel needs of electric vehicle owners before leaving work and improve owner satisfaction, the electric vehicle is guided to start charging by reducing the dynamic electricity price. With the access of a small number of residential electric vehicles, the electricity price is appropriately increased at 20:00 to suppress the centralized charging of electric vehicles in surrounding residential areas. Between 21:00 and 3:00, the charging of electric vehicles is guided by reducing the dynamic electricity price.

In summary, by means of dynamic electricity price, electric vehicles are guided to discharge during the concentrated period of the original load, charge during the low period of the original load, and hedge with the load of the distribution network, which helps to alleviate the conflict of interest among the owners of the charging station. Compared with the fixed time-of-use electricity price, the dynamic electricity price is more flexible.

In order to verify the performance of the optimal scheduling model of electric vehicles in this paper, the following scenes are used for comparative experiment:

The *F*_1_ target is that the electric vehicle charging station has the largest revenue, the *F*_2_ target is that the owner comprehensive satisfaction is the highest, and the *F*_3_ target is that the peak-valley difference rate of electric vehicle load is the lowest.

Scene 1: Disorderly charging of electric vehicles, that is, the scheduling arrangement of electric vehicle charging stations is not accepted. After the owner arrives at the charging station, the electric vehicle is immediately connected to the power grid for charging, and the charging and discharging process is not optimized.

Scene 2: The electric vehicle charging station uses the time-of-use price in Table 1 to guide and optimize the charging and discharging of electric vehicles. The optimization objectives are *F*_1_, *F*_2_ and *F*_3_ in this paper.

Scene 3 (method in this paper): The electric vehicle charging station adopts the dynamic electricity price guidance method in this paper to schedule and optimize the charging and discharging of electric vehicles. The optimization objectives are *F*_1_, *F*_2_ and *F*_3_ in this paper.

Scene 4: The electric vehicle charging station adopts the dynamic electricity price guidance method in this paper to optimize the scheduling of electric vehicle charging and discharging. The optimization goal are *F*_1_,*F*_3_ and the comprehensive satisfaction of *F*_2_ without considering *α*_n1_ (Economic satisfaction).

Scene 5: The electric vehicle charging station adopts the time-of-use electricity price guidance method in this paper to optimize the scheduling of electric vehicle charging and discharging. The optimization goal are *F*_1_, *F*_3_ and the comprehensive satisfaction of *F*_2_ without considering *α*_n1_ (Economic satisfaction).

**Fig 12:**
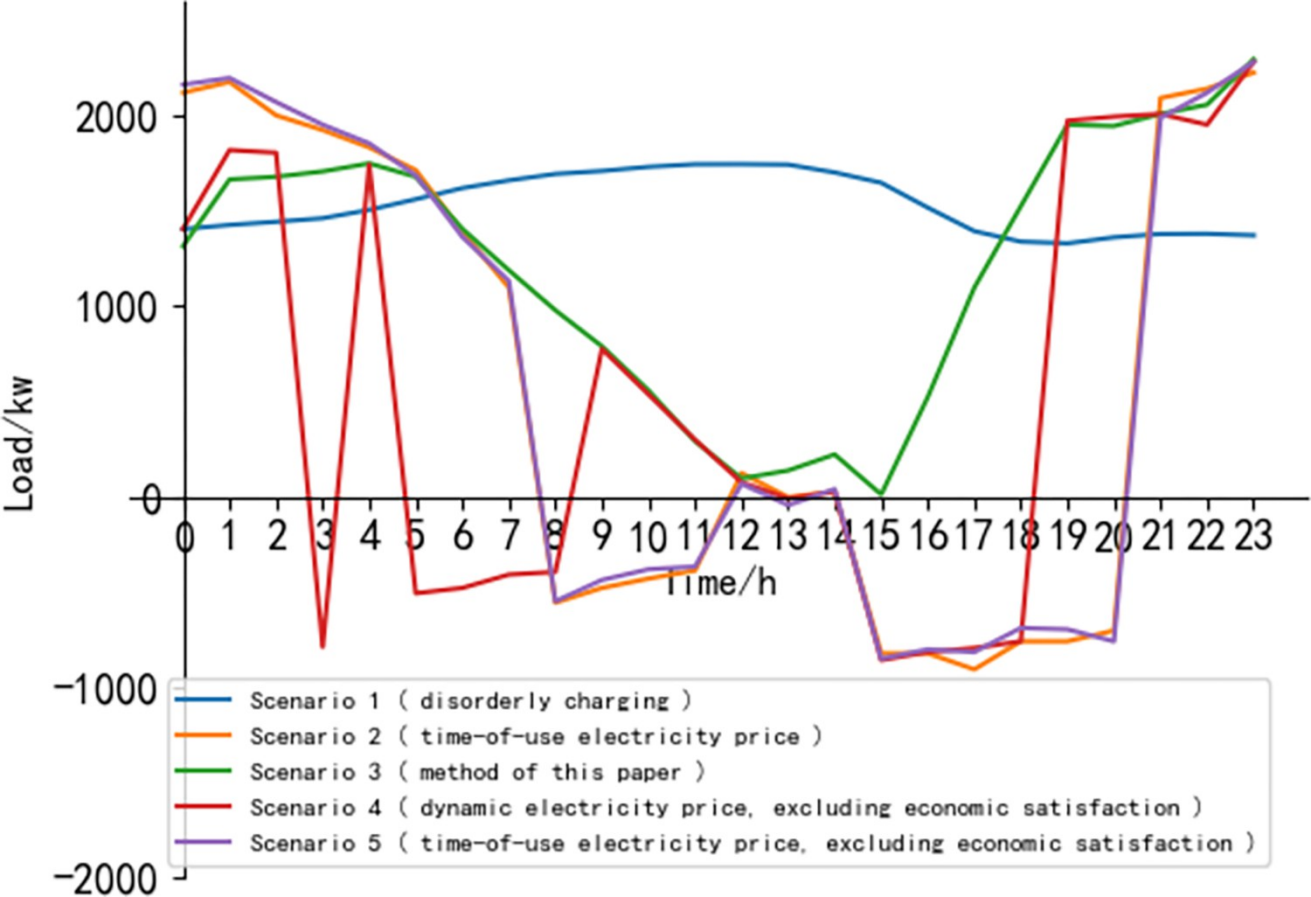
Electric vehicle load curve under different scenes.

Fig 12 shows the comparison of charging and discharging loads of electric vehicles solved by DJM-NSGA-III algorithm in different scenarios. Among them, the time complexity of scene 3 is the highest, and the time complexity of scene 4 and scene 5 is the second and the difference is small. In daily life, the load of the park’s charging station will gradually increase as employees go to work. Electric vehicles are concentrated during work, and as a large number of electric vehicles complete charging and employees leave work, the load gradually decreases. Scene 3 is the method used in this paper. The overall trend shows that the electric vehicle load is mainly concentrated in the two periods of 0:00–6:00 and 18:00–23:00. The charging station dispatches the electric vehicle to charge in these periods to avoid the peak load period during the day. At 6:00–12:00, the charging station guides the electric vehicle to discharge at this time by increasing the electricity price, improving the interests of the owner and feeds the distribution network to alleviate the load pressure of the distribution network. The load gradually increases after 15:00. At this time, in order to meet the demand for electric vehicle power before work, the charging capacity of electric vehicles gradually increases, and the load shows a rapid upward trend. As employees leave work, the growth rate slows down after 19:00, but with the access of electric vehicles to surrounding residents, the load rises slowly.

The following is an overview of each scene:

In scene 1, the charging and discharging process is not optimized, so the load is concentrated during the employee’s work. At 0:00–7:00, the charging load is on the rise as a whole. Most of the charging load is concentrated between 08:00 and 15:00, that is, the peak period of charging. Between 16:00 and 18:00, the load shows a downward trend, and tends to be gentle after 19:00. In scene 2, the charging and discharging decision of electric vehicles is affected by time-of-use electricity price, which is concentrated in the peak period of charging at work, charging in the morning and afternoon, resulting in large load fluctuation. Scene 3 (the method in this paper) adopts dynamic electricity price guidance, and its load fluctuation is slowed down compared with other scenes. Scene 4 does not consider the economic satisfaction of the owner, and the charging station scheduling leads to severe load fluctuations in order to maximize its own revenue. Scene 5 adopts time-of-use electricity price, and the economic satisfaction of the owner has little effect on the load of the electric vehicle. The load curve is similar to that of scene 2, but it is slightly higher in some periods.

On the whole, the charging and discharging scheduling strategy of electric vehicles in this paper can effectively disperse the charging demand, avoid the peak load pressure, provide power for the distribution network, and realize the intelligent coordination of electric vehicle charging and grid load, which is more flexible than the traditional scheduling method.

Table 3 shows the comparison of the objective function values in different scenes. Among them, the income side of the charging station is scaled by percentage due to the large gap between the value and the other two objective function values in the previous solution, and the intuitive display is restored here. Scene 1 is a disorderly charging situation. After the owner arrives at the charging station, the electric vehicle is immediately connected to the power grid for charging, and the charging and discharging process is not optimized. Therefore, the load fluctuation caused by discharge will not occur, and the load peak-to-valley difference rate is the lowest. The load peak-to-valley difference rate of the other four scenes has been improved. In order to achieve the purpose of hedging with the regional distribution network load and alleviating the regional distribution network load, the discharge scheduling of the other four scenes has generated a new load valley value. Compared with scene 2 (time-of-use electricity price), the targets under scene 3 (method in this paper) have been improved. The income of electric vehicle charging station has increased by 32500 yuan, the comprehensive satisfaction of vehicle owners has increased by 2%, and the load peak-valley difference rate has decreased by 0.41, which effectively slows down the load peak-valley difference rate, alleviates the conflict of interest between vehicle owners and charging stations, and achieves a win-win situation. Compared with scene 4 (dynamic electricity price without considering the economic satisfaction of the owner), the income of the electric vehicle charging station in scene 3 (the method in this paper) is increased by 8500 yuan, the satisfaction of the owner is basically the same, and the peak-to-valley difference of the load is low by 0.4, indicating that considering the economic satisfaction of the owner under the dynamic electricity price policy can guide the owner to actively participate in the charging and discharging scheduling, alleviate the severe load fluctuation, and improve the income of the charging station. Scene 5 uses time-of-use electricity price and does not consider the economic satisfaction of the owner. Compared with scene 4, the revenue of the charging station is 27,000 yuan lower, the satisfaction of the owner is 9% higher, and the peak-valley difference rate is basically the same, indicating that although the use of dynamic electricity price sacrifices the satisfaction of some owners, it can effectively improve the revenue of the charging station.

**Table 3:** Comparison of the objective function values under different scenes.

	Owner satisfaction/%	Charging station revenue / yuan	Load peak-valley difference rate
Scene 1	56%	22000	0.3
Scene 2 (Time-of-use electricity price)	59%	10500	1.22
Scene 3 (Method in this paper)	61%	43000	0.81
Scene 4 (not considered *α*_n1_)	62%	34500	1.21
Scene 5 (Time-of-use electricity price, not considered *α*_n1_)	71%	7500	1.22

## 7 Conclusion

In order to alleviate the conflict of interest between the electric vehicle charging station and the owner and prevent the load fluctuation caused by the centralized charging of large-scale electric vehicles, this paper proposes an optimal scheduling strategy for electric vehicles considering the comprehensive satisfaction of the owner. An electric vehicle scheduling model with the optimization objectives of maximizing the revenue of electric vehicle charging stations, maximizing the comprehensive satisfaction of vehicle owners’ economy and minimizing the peak-valley difference rate of distribution network load is constructed, and the DJM-NSGA-III algorithm is used to solve the model. The simulation results show that the scheduling strategy proposed in this paper can effectively improve the income of electric vehicle charging stations and the satisfaction of vehicle owners, alleviate the conflict of interest between electric vehicle charging stations and vehicle owners, and achieve a win-win situation. Through the dynamic electricity price, the electric vehicle charging and discharging scheduling is reasonably guided, so that the electric vehicle load and the distribution network load are hedged, and the power supply pressure of the distribution network is reduced.

In addition, with the rise of clean energy such as photovoltaic and wind power, the future can further study how to coordinate the operation of electric vehicle charging stations with photovoltaic and wind power, maximize the use of renewable energy, reduce grid dependence, and promote sustainable development.

## References

[1] Yu CUI, Wenjiang LIU. Development and promises of li-ion power battery technology for new energy vehicles[J]. Journal Of Northeast Electric Power University, 2022, 42(2): 41–48.

[2] Bai XIAO, Jiaxun ZHU, Xin LIU, et al. Bi-level Planning of Electric Vehicle Charging Stations Based on CRITIC Method and Non-cooperative Game[J]. Journal Of Northeast Electric Power University, 2022, 42(04): 35–49.

[3] Meghana Pulimamidi and Yammani Chandrasekhar and Salkuti S. Blockchain technology based decentralized energy management in multi-microgrids including electric vehicles Journal of Intelligent & Fuzzy Systems (2022):42.

[4] Mohanty A. K. and Babu P. S. and Salkuti S. Fuzzy-Based Simultaneous Optimal Placement of Electric Vehicle Charging Stations, Distributed Generators, and DSTATCOM in a Distribution System Energies (2022).

[5] Zitong Zhao, Bing Gu. Research on Charging and Discharging Price and TimePeriod based on Electric Vehicle Load Aggregator under Demand Response [J]. Journal Of Northeast Electric Power University, 2023,43(06):79–86. doi: 10.13335/j.1000-3673.pst.2016.04.024

[6] Cuiping LI, Wenchao ZHU, Junhui LI, et al. Research on the Operation Schemeof Distributed Generation Access to Medium Voltage Distribution Network [J]. Journal Of Northeast Electric Power University,2023,43(4):57–64.

[7] Zhongzhi PAN, Ning KONG, Yantao WANG. Joint Optimal Allocation Method of Energy-Load-Storage in Power Supply Area to Improve New Energy Consumption Capacity [J]. Journal Of Northeast Electric Power University, 2023,43(06):71–78. doi: 10.19718/j.issn.1005-2992.2023-06-0071-08

[8] Guihong Huang, Xin Lei, Yi Yang, et al. Two-layer Smart Charge-Discharge Strategies for Electric Vehicles Considering Wind Generation and Users’ Satisfaction[J]. TRANSACTIONS OF CHINA ELECTROTECHNICAL SOCIETY, 2015, 30(05): 85–97.

[9] Yan DONG, Aolong WEI, Yongsheng ZHU, et al. Research on bi-level charge and discharge dispatching of electric vehicle considering user satisfaction degree[J]. JOURNAL OF ZHONGYUAN UNIVERSITY OF TECHNOLOGY, 2020, 31(03): 63–70.

[10] Tianyi Han, Xi Chen, Qingxin Liu. Short-Term Load Forecasting for Distribution Network in The Presence of TOU Price Based on Long-Short-Term Memory Network[J]. Journal Of Northeast Electric Power University, 2020, 40(4): 19–28.

[11] Lupeng CHEN, Zhenning PAN, Tao YU, et al. Dynamic Coordination Mechanism of Grid Frequency Regulation with Multiple Photovoltaic Generation Units[J]. Automation of Electric Power Systems, 2019, 43(24): 32–40+66.

[12] Jindong CUI, Wenda LUO, Niancheng ZHOU. Research on Pricing Model and Strategy of Electric Vehicle Charging and Discharging Based on Multi View[J]. Proceedings of the CSEE, 2018, 38(15): 4438–4450+4644.

[13] Xizhu ZHANG, Xunyuan LIU, Wentao YANG, et al. A Hierarchical Scheduling Strategy for Electric Vehicles under Dynamic Time-of-use Tariff Mechanism[J]. Electric Power Construction, 2018, 39(12): 73–80.

[14] ZHOU C, QIAN K, ALLAN M, et al. Modeling of the cost of EV battery wear due to V2G application in power systems[J]. IEEE Transactions on Energy Conversion, 2011, 26(4): 1041–1050.

[15] Haolin WANG, Yongjun ZHANG, Haipeng MAO. Charging load forecasting method based on instantaneous charging probability for electric vehicles[J]. Electric Power Automation Equipment, 2019, 39(03): 207–213.

[16] XIAO Li, Yaoping XIE, Huafeng HU, et al. Two-level Optimization Scheduling Strategy for EV’s Charging and Discharging Based on V2G[J]. Electric Power Automation Equipment, 2019, 39(03): 207–213.

[17] Yiling CHEN, Zhipeng LÜ, Shan ZHOU. Multi-objective optimal scheduling of electric vehicles based on cloud edge end cooperation[J] Distribution \& Utilization, 2022, 39(04): 17–24.

[18] Yuling Luo. Research on the Prediction Method of Charging Power Based on the Behavior of Electric Vehicle Owners [D]. Xi’an Technological University, 2022.

[19] Fengjiao YU, Dian WANG, Runyu LI. Optimization Strategy for Grid Connection of Distributed Generation Considering Demand Side Response[J]. Journal Of Northeast Electric Power University, 2022,42(02):92–103.

[20] Lijuan Geng. Research on electric vehicle scheduling strategy based on V2G[D]. Qingdao University, 2016.

[21] Pu SUN, Hao LIU, Yong ZHANG, et al. An improved atom search optimization with dynamic opposite learning and heterogeneous comprehensive learning[J]. Applied soft computing, 2021, 103: 107140.

[22] Zhanbo YANG, Ping MA. Reactive power optimization of distributed power distribution system based on Jaya algorithm[J]. Electronic Design Engineering2023,31(08):143–146+151.

[23] Xiaoming SUN Wei WANG, Su SU, et al. Coordinated Charging Strategy for Electric Vehicles Based on Time-of-use Price[J]. Automation of Electric Power Systems, 2013,37(01):191–195.

[24] Zihao LIU, Baoliang DONG, Baobao WANG, et al. Model for conventional missile firepower distribution based on R-NSGA-III[J]. Electronic Design Engineering,2022,30(23):169–173.

[25] Sumetpipat K, Baowan D. Stable Configurations of DOXH Interacting with Graphene: Heuristic Algorithm Approach Using NSGA-II and U-NSGA-III[J]. Nanomaterials, 2022, 12(22): 4097. 36432383 10.3390/nano12224097 PMC9693072

